# Versatile soft X-ray-optical cross-correlator for ultrafast applications

**DOI:** 10.1063/1.4964296

**Published:** 2016-10-11

**Authors:** Daniel Schick, Sebastian Eckert, Niko Pontius, Rolf Mitzner, Alexander Föhlisch, Karsten Holldack, Florian Sorgenfrei

**Affiliations:** 1 Institut für Methoden und Instrumentierung der Forschung mit Synchrotronstrahlung, Helmholtz-Zentrum Berlin für Materialien und Energie GmbH, Albert-Einstein-Straße 15, 12489 Berlin, Germany; 2 Institut für Physik und Astronomie, Universität Potsdam, Karl-Liebknecht-Straße 24-25, 14476 Potsdam, Germany

## Abstract

We present an X-ray-optical cross-correlator for the soft (>150 eV) up to the hard X-ray regime based on a molybdenum-silicon superlattice. The cross-correlation is done by probing intensity and position changes of superlattice Bragg peaks caused by photoexcitation of coherent phonons. This approach is applicable for a wide range of X-ray photon energies as well as for a broad range of excitation wavelengths and requires no external fields or changes of temperature. Moreover, the cross-correlator can be employed on a 10 ps or 100 fs time scale featuring up to 50% total X-ray reflectivity and transient signal changes of more than 20%.

## I. INTRODUCTION

Time-resolved X-ray experiments allow to access a wide variety of transient microscopic processes on the relevant time-scales, e.g., in molecular dynamics,^1^ magnetism^2^ or lattice dynamics in solids.^3^ In order to probe individual degrees of freedom in a selective manner, it is often necessary to tune the pump and/or probe wavelengths with respect to element-specific resonances. While for certain fixed X-ray energies, techniques based on reference samples exist, the wide range of X-ray wavelengths that are of interest and the many experimental techniques used, such as scattering, absorption, or photoelectron spectroscopy, call for flexible cross-correlation techniques to precisely overlap the pump and probe pulses in time and space. For practical reasons, the cross-correlator should ideally be adaptable to varying experimental settings in order to avoid time-consuming changes of the setup possibly causing shifts of the temporal and spacial overlap of the pump and probe pulses. To reduce expenditure of time for re-checking overlap during a running measurement campaign, a high signal-to-noise ratio is inevitable. Especially at SASE free electron lasers (FELs), the shot-to-shot jitter already led to the development of single-shot cross-correlation techniques^4^ which require comparably high X-ray flux for pumping a reference sample. For low and moderate X-ray fluxes, laser-excited processes can be employed for cross-correlation, e.g., the coherent excitation of optical phonons in bismuth^5,6^ or acoustic phonons in superlattice (SL) structures^7,8^ probed by hard X-ray pulses.

**FIG. 1.**
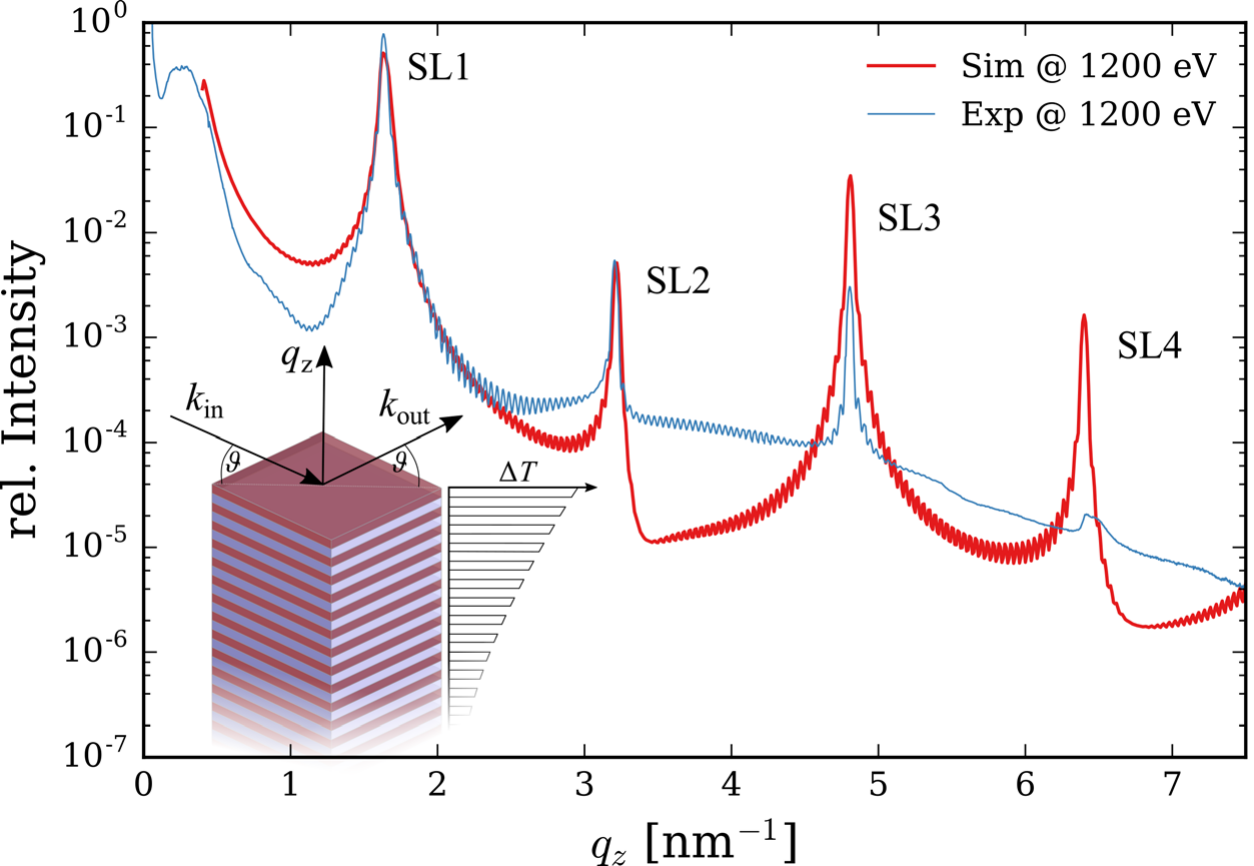
Static ϑ/2ϑ-scan along *q_z_* of the Mo/Si SL at $E_{\text{ph}}=1200\;\text{eV}$ (thin blue line) in a specular reflection geometry carried out at the PM3 beamline at BESSY II. The dynamical X-ray diffraction simulation of the SL structure (thick red line) matches the SL's peak positions and the reflectivity for the first and second order SL peaks (SL1 and SL2). The inset sketches the SL structure after photoexcitation with an exponentially decaying periodic temperature profile, as well as the symmetric diffraction geometry.

In the soft X-ray range, structural dynamics is hardly accessible since here the X-ray wavevector is small compared to the reciprocal lattice constants. In a specular reflection geometry, the momentum transfer *q_z_* is given by

$$q_{z}=\frac{4\pi }{\lambda }\text{sin}\left(\vartheta \right),$$ (1)

where *λ* denotes the X-ray wavelength and ϑ the angle between incoming/outgoing X-ray wavevectors $\vec{k}$ and the sample surface, see inset in Fig. 1. In specific experimental constellations, time-resolved X-ray magnetic circular dichroism (XMCD) experiments on magnetic samples can serve as a handy cross-correlator probing, i.e., the ultrafast demagnetization after photoexcitation.^2^ Such experiments require the X-ray photon energy tuned to the respective electronic resonance, elliptically polarized X-rays, as well as an external magnetic field (and possibly sample cooling) to bring the sample back into a magnetic state after each laser excitation. Obviously such techniques are only suited for special cases and most experiments call for a more flexible cross-correlation method especially for the soft X-ray range.

In this work, we adapt the concept of von Korff Schmising *et al.* to photo-excite coherent longitudinal acoustic phonons in a superlattice (SL) structure. Here the SL is a mirror for the whole soft X-ray regime for photon energies >150 eV consisting of alternating silicon and molybdenum layers of a few nanometer thickness.^9,10^ The artificial superstructure of this cross-correlator gives access to photoexcited structural dynamics in the soft up to the hard X-ray regime and for a broad range of laser excitation wavelengths. The simple ϑ/2ϑ setup of this laser-pump X-ray-probe measurement requires only a variable delay between the two pulses and an X-ray sensitive detector, while no external fields or temperature changes are necessary. Two types of lattice dynamics are observable, which allow for addressing both the 10 ps and 100 fs time scale depending on the temporal resolution of the experiment.

## II. EXPERIMENTAL RESULTS AND DISCUSSION

In the case presented here, the investigated SL structure consists of 40 bilayers made of $d_{\text{Si}}=2.07\;\text{nm}$ amorphous silicon and $d_{\text{Mo}}=1.86\;\text{nm}$ polycrystalline molybdenum grown on crystalline silicon (AXO Dresden GmbH, Dresden, Germany). This results in a bilayer thickness of $d_{\text{SL}}=3.9\;\text{nm}$ and a corresponding reciprocal lattice vector of $G_{\text{SL}}=1.61\;{\text{nm}}^{-1}$. Static ϑ/2ϑ-scans for photon energies $E_{\text{ph}}$ from 200 up to 2000 eV were carried out at the PM3 beamline^11^ at BESSY II. A representative scan at $E_{\text{ph}}=1200\;\text{eV}$ is shown in Fig. 1 revealing several orders of the SL Bragg peak (marked as SL*n*) at $q_{z}^{n}=n\;G_{\text{SL}}\;(n\in \mathbb{N})$.

The dynamical X-ray diffraction simulation^12^ of the static structure matches the SL Bragg peak position and allows for precise structure refinement. The discrepancy between the measured and simulated peak intensities for the SL3 and SL4 peaks can be attributed to small interdiffusion layers of MoSi_2_ between the Mo and Si layers in the SL structure^10^ and additional surface roughness of the nanostructure which have been neglected for simplicity. The SL1 Bragg peak of the current Mo/Si SL is accessible down to $E_{\text{ph}}=150\;\text{eV}$ with a scattering geometry close to normal incidence. This SL has been optimized for higher X-ray photon energies between 400 to 1200 eV with a reflectivity between 10% to 50%. For smaller X-ray energies, SL structures with larger bilayer thickness can be fabricated with more than 70% reflectivity around $E_{\text{ph}}=100\;\text{eV}$.^9^ The SL2 reflection of the current structure is accessible down to 317 eV and the SL3 reflection down to 476 eV. Both Bragg peaks diffract between $10^{-3}-10^{-2}$ of the incident X-ray photons resulting in reasonable detectability for most pulsed X-ray sources.

The time-resolved experiments with either 75 ps (normal single bunch^13,14^) or 120 fs (laser slicing; all pulse lengths are given as FWHM) temporal resolution have been carried out at the FemtoSpeX facility (UE56/1 ZPM) at BESSY II.^14–16^ The Mo/Si sample was excited by 50 fs laser pulses of 800 nm central wavelength at 3 kHz repetition rate. The measurements were all carried out in a ϑ/2ϑ geometry with a fast avalanche photo diode (APD). The setup allowed for easy switching between analogue acquisition mode (with the APD in a proportional operation mode) or photon counting acquisition mode (with the APD in a Geiger mode) depending on the probed SL order and the incoming X-ray flux of the beamline.

When the Mo/Si SL is excited by a fs laser pulse, the crystal lattice of all metallic molybdenum layers is quasi-instantaneously heated with only the electron-phonon coupling time as time lag. The amorphous silicon SL layers and the crystalline silicon substrate are basically transparent for the 800 nm pump laser pulses. The resulting spatial excitation profile has the same periodicity as the SL itself multiplied with an amplitude exponentially decaying on the pump wavelength absorption length according to Lambert-Beer's law, see inset in Fig. 1. Here the application of Lambert-Beer's law, which does not take internal reflections into account, is valid since interference effects can be neglected due to the smaller SL period and total SL thickness compared to the pump laser wavelength. Other derivations from a transfer-matrix formalism^17^ in the actual absorption profile can be compromised by scaling the excitation laser fluence. The photoinduced thermal stress from electron phonon-coupling and possibly the stress from deformation-potential interaction of the hot electrons is then released via an initial expansion of the molybdenum layers and a simultaneous compression of the silicon layers while preserving the SL period $d_{\text{SL}}$. This is true for a broad range of optical pump wavelengths from the mid-IR to the UV for which the photoinduced stress in the molybdenum layers is much larger than in the silicon layers. Like an optical phonon, this initial lattice motion then reverses and starts thickness oscillations of all bilayer constituents around a new equilibrium position due to the displacive photoexcitation of the SL structure.^18^

**FIG. 2.**
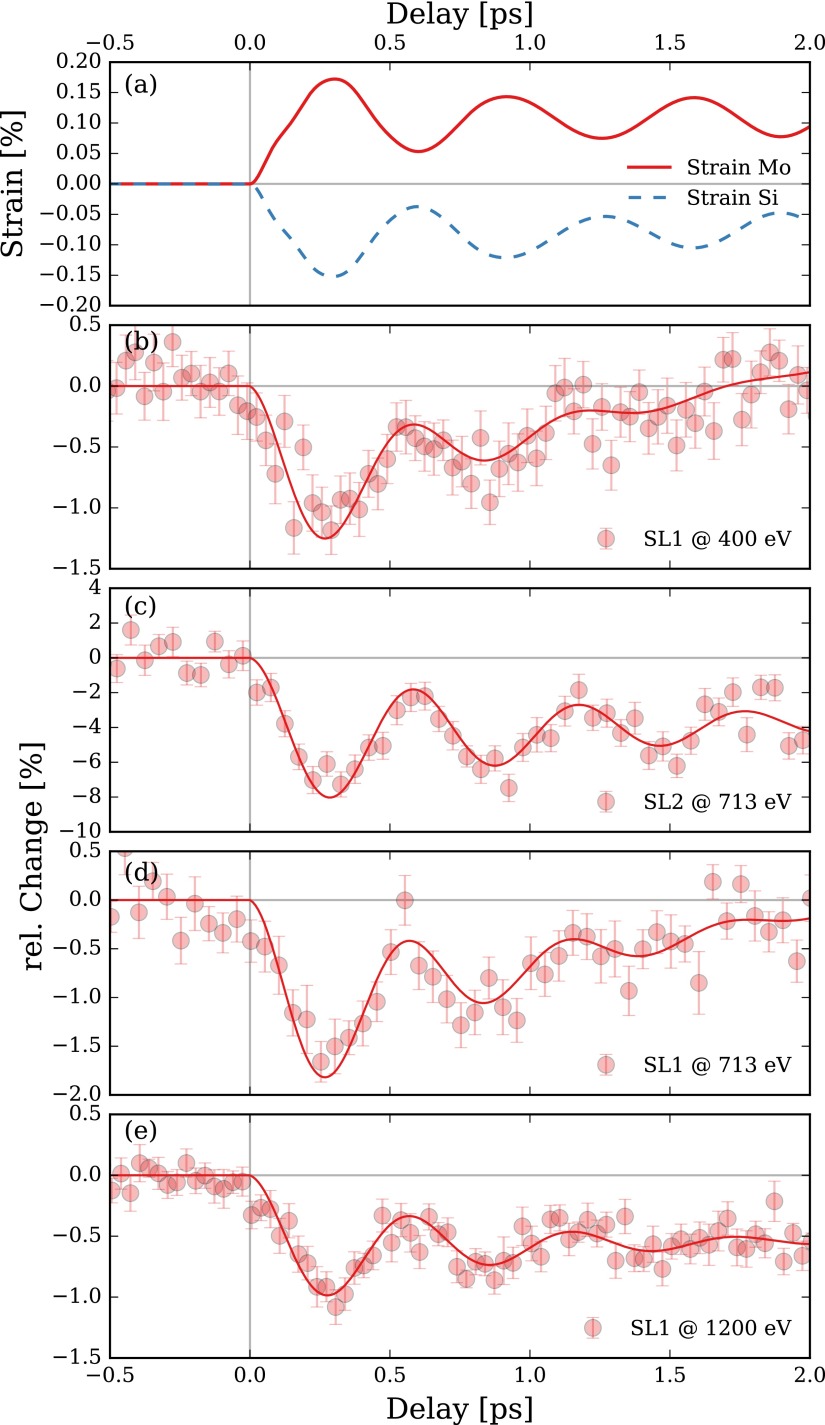
(a) Simulated transient strain of only the Mo and Si layers in the SL structure after photoexcitation. (b)-(e) SL oscillation measured at SL Bragg peak maxima at different X-ray photon energies, see legends, with 120 fs temporal resolution in femtoslicing mode. Symbols are measured data and solid lines are fits. The errorbars are at a 66% confidence interval.

These oscillations can also be described in terms of reflection and transmission of coherent acoustic phonons at each SL interface. In order to simulate such lattice dynamics, we apply a simple one-dimensional masses-and-spring model.^12,19^ We assume an instantaneous photoexcitation of coherent acoustic phonons in the Mo layers and completely neglect heat diffusion for simplicity. The resulting spatio-temporal strain matrix for the SL structure can be further used as an input for transient dynamical X-ray diffraction calculations to fully simulate the experimental response of the SL after laser excitation.^12^ The spatially averaged strain of the Mo and Si layers in the current SL are shown in Fig. 2(a) and reveal an oscillation period of $P_{\text{SL}}\approx 600\;\text{fs}$ using literature values for the thermoelastic material properties.

We quantified the sub-ps phonon dynamics of the current Mo/Si SL at the FemtoSpeX facility at BESSY II in slicing mode with approximately 120 fs temporal resolution by probing intensity oscillations of different SL Bragg peaks. These intensity oscillations are caused by the quasi-optical phonon mode of the SL structure and can also be described as transient changes of the structure factor of a single SL bilayer.^7,8,20^ The experimental data of these measurements are shown in Figs. 2(b)–2(e) as symbols and the solid lines represent cosine-fits. For the SL1 peak, the transient oscillation amplitude is rather small with only 1%, but the high reflectivity of this peak for the complete X-ray photon energy range investigated here (400 to 1200 eV) still allows for convenient cross-correlation, see Figs. 2(b), 2(d), and 2(e). The SL2 peak shows nearly 10% oscillation amplitude, cf Fig. 2(c), but at nearly 100 times smaller reflectivity than the SL1 peak. The oscillation period $P_{\text{SL}}$ of the experimental fits vary between 573 to 611 fs and are in good agreement with the simulated value of $P_{\text{SL}}=600\;\text{fs}$ shown in Fig. 2(a). A representative measurement of the SL oscillation at, i.e., 713 eV on the SL1 peak, see Fig. 2(d) took about 30 min and is hence still reasonably fast for flexible cross-correlation on the fs timescale.

The precision of determining the temporal overlap between the pump and probe pulses is limited by several factors. The intrinsic time scale of the probed dynamics serves as an upper limit for the time zero determination, which is approximately 600 fs for the investigated SL. If the experimental response of the cross-correlator is well known, a corresponding fit function can be used to determine time zero with much higher precision that is in principle only limited by the statistics of the data. For the SL presented here, the cosine-fits allow for a very precise determination of the oscillation onset with ±15 fs and hence a very good repeatability of this specific pump-probe delay.

**FIG. 3.**
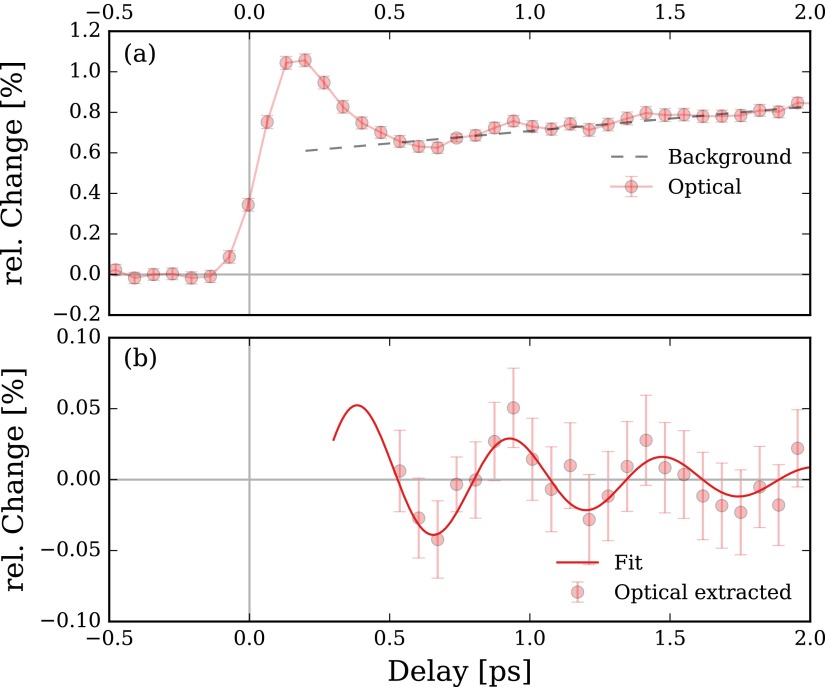
(a) Transient optical SL reflectivity. The gray dashed line is subtracted as background in (b), which shows only the extracted SL oscillation. The solid line represents a damped cosine fit.

However, as discussed in Refs. 7 and 8, the exact temporal overlap between pump and probe pulses can in principle deviate from the *t*_0_ of the cosine fits due to the electron-phonon coupling time in the Mo layers in the SL structure. In order to experimentally narrow down the electron-phonon coupling time, we carried out time-resolved all-optical Brillouin scattering experiments on the same SL.^8,21^ The all-optical data shown in Figs. 3(a) and 3(b) has been measured with 50 fs pump and probe pulses at 800 nm wavelength in a ϑ=45° reflection geometry. The incident excitation fluence was set to approximately $20\;\text{mJ}\;{\text{cm}}^{-2}$. The optical reflectivity in Fig. 3(a) shows a steep rising slope due to initial electronic heating of the Mo limited by the temporal resolution of the setup. The steepest slope determines the temporal overlap between pump and probe pulses and is set to *t* = 0. The following plateau due to heating of the lattice features a small oscillation of the reflectivity which has been extracted by background subtraction in panel (b). The solid line in this panel corresponds to a cosine-fit with an oscillation period of 575 fs and can be obviously linked to the coherent phonon oscillation in the SL structure with the simulated oscillation period of $P_{\text{SL}}=600\;\text{fs}$. The fast onset of the extracted optical reflectivity oscillations allows to determine the electron-phonon coupling time in Mo to be faster than 500 fs. The observed oscillation can be explained as interference of the probe light from the SL surface and the coherent acoustic strain fronts in the SL. Since this effect sensitively depends on the actual probe wavelength, it is not possible to directly link the absolute phase of the optically observed oscillation to the underlying lattice motion shown in Fig. 2(a).^8^ Hence the all-optical data alone does not allow to determine the absolute time zero of the Mo/Si SL oscillation shown in Figs. 2(b)–2(e) below the estimated upper limit of the electron-phonon coupling time of 500 fs. Alternatively, we calibrated the absolute time zero of the femtoslicing X-ray experiment with an ultrafast XMCD experiment probing the laser-induced demagnetization in a ferromagnet to be correct within ±500 fs. More precise calibrations are planned for upcoming experiments.

**FIG. 4.**
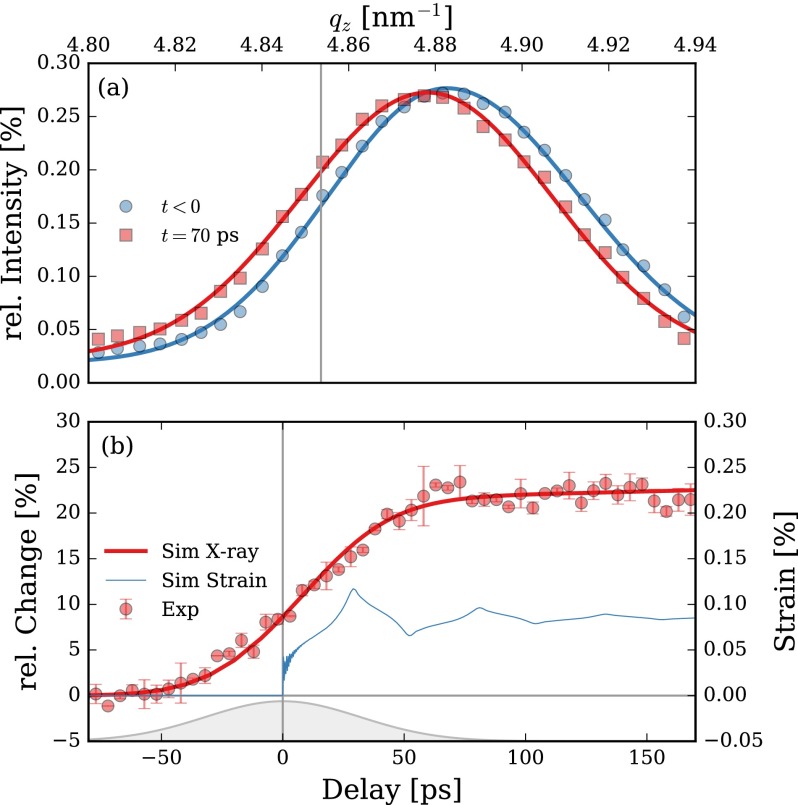
(a) Unpumped ϑ/2ϑ-scan at 713 eV around SL3 and probed 70 ps after excitation with a laser fluence of $F=12\;\text{mJ}\;{\text{cm}}^{-2}$ with 75 ps temporal resolution in single-bunch mode. The shift of the SL Bragg peak corresponds to a strain of 0.09% averaged over the whole probe SL volume. (b) Delay scan (symbols, left y-axis) at the left slope at $4.853\;{\text{nm}}^{-1}$ of the SL3 peak at 713 eV [marked as vertical line in (a)] in single-bunch mode. The errorbars are at a 66% confidence interval. The thin blue line (right *y*-axis) represents the simulated averaged strain in the Mo/Si SL. The thick red line (left *y*-axis) is the calculated X-ray reflectivity at $q_{z}=4.853\;{\text{nm}}^{-1}$ based on the simulated strain and convoluted with the temporal resolution of 75 ps (gray Gaussian at the bottom) of the single bunch at BESSY II.

For later delay times, after the SL oscillations have damped out due transmission of the coherent phonons to the substrate, the entire SL structure remains in an expanded state until heat diffusion subsequently leads to thermal equilibrium between the Mo and Si layers in the SL and to cooling of the SL due to thermal transport into the substrate. The later thermal process occurs on a ns-timescale^22,23^ and thus does not affect the probed transient reflectivity changes presented here. The maximum laser-induced expansion of the whole SL structure can be calculated from the lattice dynamics simulation described above.^12^ The spatially averaged strain of all Mo and Si layers in the SL from these simulations is shown in Fig. 4(b) and reaches its maximum after 28 ps. The subsequent small oscillation of the spatially averaged strain in the SL is due to the rather bad matching of the acoustical impedance between the Mo and Si in the SL and substrate.

The total expansion of the SL can be directly probed by the shift of each SL Bragg peak in time-resolved ϑ/2ϑ scans. The upper panel in Fig. 4 depicts a ϑ/2ϑ-scan of the SL3 probed at $E_{\text{ph}}=713\;\text{eV}$ with 75 ps temporal resolution (single-bunch) before photoexcitation (*t* < 0) and 70 ps after photoexcitation. The incident laser fluence was set to approximately $F=12\;\text{mJ}\;{\text{cm}}^{-2}$ at 800 nm wavelength. The acquisition of the two scans took only 16 s integration time in total. The significant shift between the unpumped and pumped SL Bragg peaks is the result of ε=0.09 % expansive strain in the probed SL volume at late delays which can be directly deduced from the peak shift $\Delta q=0.004\;{\text{nm}}^{-1}$. Although the relative Bragg peak shift is the same for all SL orders, the absolute shift is proportional to the SL peak position $q_{z}^{n}$, which results in stronger intensity changes at the Bragg peak slopes for higher SL orders for the same laser-induced strain. We selected $q_{z}=4.853\;{\text{nm}}^{-1}$ on the left slope of the SL3 Bragg peak which shows reasonable reflectivity and a strong transient change to carry out a delay scan, shown in Fig. 4(b) as red symbols, which took only 80 s of the total integration time. The transient increase of diffracted X-ray intensity by more than 20% is directly linked to the SL Bragg peak shift (peak broadening can be neglected here). The probed dynamics are limited by the 75 ps temporal resolution of the single-bunch of BESSY II and can be well reproduced by taking the simulated transient strain (solid blue line) as input for dynamical X-ray diffraction calculations.^12^ Similar to the experiment, we extracted the relative diffracted X-ray intensity at $q_{z}=4.853\;{\text{nm}}^{-1}$ from the X-ray simulations and convoluted it with the 75 ps temporal resolution of the real experiment showing nearly perfect agreement with the experimental data, cf. red solid line in Fig. 4(b).

The underlying 10 ps time scale renders this transient peak shift an ideal cross-correlator for ps-pulsed X-ray sources like the normal single bunch of 3rd generation synchrotrons (approximately 75 ps at BESSY II),^13,14^ synchrotrons in low-*α* mode (approximately 10 ps at BESSY II)^24^ and even below that such as for the currently developed BESSY-VSR mode.^24^ Moreover, the ns recovery of the peak shift yields efficient detectability of this transient signature and can also be utilized for optimizing the spatial overlap between pump and probe pulses since the transient signal change is proportional to the excitation fluence in the probed region. We have verified the feasibility of the SL Bragg peak shift on the ps time scale for different X-ray photon energies between 400 to 1200 eV and in addition to 800 nm also at 266 nm pump laser wavelength which all showed similar results as presented in Fig. 4.

## III. CONCLUSION

We presented a simple and fast cross-correlation method for the soft (E>150 eV) up to the hard X-ray regime utilizing a Mo/Si SL structure. The method probes, for the first time at the FemtoSpeX facility at BESSY II, laser-induced lattice dynamics in a SL and requires no special X-ray polarization nor any special sample environment such as temperature or external fields. The laser excitation works for a wide range of wavelengths (mid-IR to UV) for which the metallic layers in the SL are much stronger excited than the semiconducting layers. Using a wide-bandgap isolator instead of a semiconductor can extend the laser excitation range deeply into the UV enabling even shorter laser pulse durations. For the actual cross-correlation measurement, only a simple ϑ/2ϑ geometry with an X-ray sensitive detector is required while the delay between the pump and probe pulses must be adjustable to detect transient intensity changes. The fast sub-ps SL oscillations allow for determining the oscillation onset with a precision of ±15 fs while the absolute temporal overlap between pump and probe pulse was determined to be correct within ±500 fs. Upcoming calibration experiments will improve the absolute precision of the cross-correlator below 100 fs. The slower SL Bragg peak shifting on a 10 ps time scale serves as an ideal cross-correlator for ps-pulsed X-ray sources and also allows for easy optimization of the spatial overlap of pump and probe pulses. The ability to simulate the actual experimental response of the SL structure on both time scales enables future optimization and adaption of the cross-correlation to specific experimental needs. The temporal precision of the cross-correlator can be improved by decreasing the spatial SL period which on the one hand decreases the SL oscillation period but on the other hand limits the accessibility of the SL Bragg peaks to higher X-ray photon energies and vice versa. The X-ray reflectivity of the SL can be improved for selected photon energies by exchanging the SL constituents in order to hit certain element-specific X-ray resonances of one of the materials. We are confident, that such commercially available SL structures for the soft X-ray range can serve as time-tools for the growing community of time resolved X-ray experimentalists.
